# Editorial: Vaccination strategies against ruminant infectious diseases

**DOI:** 10.3389/fvets.2023.1213269

**Published:** 2023-05-30

**Authors:** Maria Paula Del Medico Zajac, Roberto Damian Moyano, María Alejandra Colombatti Olivieri

**Affiliations:** Instituto de Agrobiotecnología y Biología Molecular (IABIMO), Instituto Nacional de Tecnología Agropecuaria (INTA), Consejo Nacional de Investigaciones Científicas y Técnicas (CONICET), Hurlingham, Buenos Aires, Argentina

**Keywords:** ruminant diseases, vaccine, control strategies, antigen production platforms, immune response, safety

Ruminant infectious diseases cause economic impact through losses in animal production and human health. Most of the commercially available veterinary vaccines are live attenuated or inactivated which induce different degrees of efficacy, i.e., decrease in clinical symptoms, pathogen dissemination, etc. (1, 2). The use of these vaccines has greatly enhanced ruminant and public wellbeing around the world, however, in some cases, they have limitations in their ability to induce protective immunity. Thus, rationally designed vaccines along with specific immunization schemes are required to achieve the desired outcome of vaccination against an infectious disease. Vaccine safety is another important consideration, not only in terms of potential risks to the target animal (to which the vaccine is administered), but also to the environment and to consumers of food derived from vaccinated animals (3).

Knowledge of the immune response induced by vaccination will contribute to identify the mechanisms involved in protection against a pathogen and would help to develop strategies to improve it. In this sense, this Research Topic reports experimental studies on the immune response and safety induced by two live-attenuated vaccines and of a rationally designed virus-like particles (VLP)-based vaccine against relevant ruminant pathogens. Also, a review discussing the efficacy of commercially available vaccines against an important respiratory virus of cattle is included.

One of the diseases affecting ruminants and causing economic losses is Johne's Disease (JD), caused by *Mycobacterium avium* subsp. *paratuberculosis* (Map). Animals are infected at an early age, mainly by the oral route. The source of infection are the feces of infected adult animals. It is a chronic disease causing diarrhea, weight loss, reduced milk production and reduced milk yield (4). Control of this disease is challenging due to the ineffectiveness of the cull and replacement strategy and the current vaccines on the market, which are based on inactivated whole-cell Map, do not provide protection against infection (5). Hanafy et al. described the safety and immune response elicited by a live attenuated vaccine candidate Map-ΔlipN (pgsN) via the subcutaneous and intranasal routes in goats, compared to the inactivated vaccine Mycopar.

Simultaneous administration of different vaccines is widely used in veterinary medicine. However, it is recommended to demonstrate the lack of interference following concurrent administration of vaccines against different pathogens. In this sense, Crawford et al. evaluate the cellular immune response of cattle immunized with either *Brucella abortus* strain RB51, a viral modified live vaccine (vMLV) against Bovine Respiratory Disease Complex or both RB51 and a vMLV vaccine. The authors measured the frequency of CD4^+^, CD8^+^, and γδ^+^ T cell populations within peripheral blood mononuclear cells (PBMCs) and the frequency of interferon gamma (IFN-γ) producing cells and demonstrated a lack of vaccine interference following concurrent administration of vMLV and RB51.

*Bos taurus papillomavirus* (BTV) causes the formation of benign skin lesions in cattle. Papillomaviruses (PVs) are small, non-enveloped, double-stranded DNA viruses, classically described as epitheliotropic. PVs are capable of infecting both humans and animals, but are host specific (6). The L1 and L2 capsid proteins play a crucial role in virus recognition by the host immune system. Therefore, they are being widely used for vaccine development. Vrablikova et al. describe the expression of capsid proteins, particularly L1, in a baculovirus system. They demonstrate that L1 proteins have the ability to generate non-infectious particles with a virus-like morphology, which achieves the activation of the immune system. This provides a platform for the production of recombinant proteins for the development of veterinary vaccines.

*Bovine respiratory syncytial virus* (BRSV) is considered one of the most important viral pathogens of the bovine respiratory disease complex (BRDC) in calves (7). BRSV-associated respiratory disease outbreaks are described in dairy and beef calves (7–9) and most commonly affects calves under 6 months of age. In order to provide information about BRSV calf vaccination to the veterinary community, Martinez et al. performed a systematic review and meta-analysis based on studies using commercially available BRSV-containing vaccines. The authors focused on studies that included experimental efficacy trials of animals under 6 months of age and analyzed morbidity and mortality rates after (the) BRSV challenge. They also studied the levels of such parameters in the presence of serum neutralizing antibodies and provided valuable information about the impact of vaccination of cattle with commercially available modified-live virus and inactivated BRSV vaccines.

In summary, this Research Topic presents different studies that provide valuable information about safety and immune response induced by a new vaccine candidate and a vaccine platform against ruminant pathogens (JD and BTV, respectively). Also, the potential effects on the immune response of cattle co-administered with RB51 and vMLV vaccines and a meta-analysis of the efficacy induced by inactivated and MLV BRSV vaccinated calves are included. We hope that the reader will find this Research Topic interesting and that it may contribute to control ruminant infectious disease by introducing new vaccine candidates.

## Author contributions

Research Topic conception, editing, and editorial writing by MD, RM, and MC. All authors contributed to the article and approved the submitted version.
